# Influencing factors of home hospice care needs of the older adults with chronic diseases at the end of life in China: a cross-sectional study

**DOI:** 10.3389/fpubh.2024.1348214

**Published:** 2024-05-27

**Authors:** Lei Wang, Yaru Li, Rui Zhao, Jiangxu Li, Xiangru Gong, Hongyu Li, Yuan Chi

**Affiliations:** ^1^College of Nursing, Jinzhou Medical University, Jinzhou, China; ^2^Department of Science and Technology, Jinzhou Medical University, Jinzhou, China

**Keywords:** home hospice, influencing factors, older adults, chronic diseases, need

## Abstract

**Introduction:**

Chronic diseases are becoming a serious threat to the physical and mental health of older people in China as their aging process picks up speed. Home hospice care addresses diverse needs and enhances the quality of life for older adult individuals nearing the end of life. To ensure the well-being of chronically ill older adults at the end of life, it is vital to explore and assess the multidimensional hospice needs of terminally ill older individuals in their homes. The aim of this study was to investigate the current situation of home hospice care needs of Chinese older adults with chronic diseases at the end of life, and to analyze the influencing factors (sociodemographic and disease-related factors).

**Methods:**

In this cross-sectional study, 247 older adult people with chronic diseases at the end of life were selected from the communities of 4 community health service centers in Jinzhou City, Liaoning Province from June to October 2023 by random sampling method. A general information questionnaire and the home hospice care needs questionnaire developed by our research group were used to investigate. Independent samples t-test or one-way ANOVA was used to compare the differences in the scores of different characteristics, and the factors with significant differences were selected for multivariate linear regression analysis to determine the final influencing factors.

**Results:**

The total score of home hospice needs of the dying older adult was 115.70 ± 12, with the mean scores for each dimension in descending order being Information Needs (3.96 ± 0.61), Social Support Needs (3.96 ± 0.44), Spiritual Needs (3.92 ± 0.43), Physical Needs (3.60 ± 0.59), Psychological Needs (3.37 ± 0.65). Status of residence, duration of illness (year), the type of disease, and self-care ability were influential factors in the total score of home hospice needs.

**Discussion:**

The need for hospice care for the terminally ill older adult is high, and healthcare professionals should implement services according to the influencing factors of need to meet their multidimensional needs and improve their quality of life.

## 1 Introduction

Chronic diseases are non-communicable diseases with a long duration and slow progression, characterized by widespread prevalence, high costs, disability and mortality (1). Since the 1990s, the most common non-communicable diseases, including stroke, ischemic heart disease, cancer and chronic obstructive pulmonary disease, have become the leading causes of death in China (2). Increasing age is the primary risk factor for the increased morbidity and mortality of most chronic diseases (3). China currently has the largest older adult population in the world, with 264 million individuals over 60 making up 18.7% of the overall population (4). It is projected that by 2050, the number of older people in China will reach 400 million, with an aging level of more than 30% (5). With the further intensification of the aging process in China, the issue of end-of-life care for the older adult with chronic diseases is imminent.

In China, most end-of-life patients prefer to spend their final stages of life at home due to limited medical resources and the traditional cultural concept of returning to one’s roots (6, 7). Since 2017, China has explicitly referred to hospice care, end-of-life care and palliative care collectively as hospice care (8). As a type of hospice service, home hospice provides palliative and supportive care to end-of-life patients and their families in their own homes by a team of community medical professionals and social volunteers (9, 10), aiming to meet the multifaceted needs of end-of-life patients and their families and optimize the quality of life of end-of-life patients. Bužgová and Sikorová confirmed that a patient’s overall quality of life can be impacted by unmet hospice needs (11). Therefore, it’s critical to evaluate the multifaceted hospice needs of terminally ill older persons in their homes in order to ensure the well-being of these individuals with chronic illnesses in their latter years. Furthermore, identifying the hospice needs of older adults with complex chronic illnesses is becoming an important concern for health systems (12).

Currently, there are many research investigations on the end-of-life needs of older adult people with cancer at home, such as the physiological need for pain control (13), the psychological need for worry and terror relief (14), the spiritual need for peace of mind (15), and other economic support (16). The complexity and intractability of the needs of senior non-cancer terminally ill patients contribute to the relative paucity of research addressing the needs of these individuals with complicated chronic multimorbidity. Most of the reported needs were related to emotional and/or psychological dimensions (social isolation, depression) (17, 18). Bajwah et al. (19) and Ryan et al. (20) both emphasize the supportive need to address clinical symptoms and maintain their activities of daily living. Older adult people with non-cancer chronic disease at the end of life are likely to experience a heavier symptom burden than those with advanced cancer, meaning that more care needs will be presented.

Western studies have reported sociodemographic and disease-related factors that primarily influence the need for home hospice care for the chronically ill older adult. The majority of research indicates women have a higher need for hospice care than men (21, 22). As older adults age, their physiologic functioning and health levels decline, which may increase the need for hospice care. Relevant research also suggests that economic level, education level, and health insurance are also influential factors in the need for hospice care (21, 23). Disease-related factors indicate that older adults with serious illnesses, such as malignant tumors, or multiple coexisting illnesses, face significant physical, psychological, and spiritual challenges, resulting in more urgent needs in all areas (23). The need for hospice care is often closely related to cultural traditions and social systems, and cultural differences between China and the West have led to differences in the factors influencing the need for home hospice care. Nevertheless, home hospice care has paid relatively little attention in China, and there has not been much research done on the variables that affect it.

This study aims to investigate the multi-dimensional hospice care needs of older adults with end-stage chronic diseases in China’s home environment, taking into account the country’s unique aging conditions. Additionally, it analyzes the sociodemographic and disease-related characteristics influencing factors to provide a basis for the provision of high-quality home hospice services.

## 2 Materials and method

### 2.1 Design and samples

This study was a cross-sectional study. A random sampling method was used to include the older adult with chronic diseases at the end of life in the communities belonging to four community health centers (providing home hospice services) in Jinzhou City, Liaoning Province, from June to October 2023, as study subjects. Inclusion criteria for end-of-life older adult: (1) age ≥ 60 years; (2) chronic non-tumor diseases, with 1 or more organs severely impaired in function, no effective means of cure into the terminal stage or patients with malignant tumors into the terminal stage (expected survival ≤6 months); (3) choose to die at home (hospice); (4) clear consciousness, with normal language communication, understanding ability. Exclusion Criteria for end-of-life older adult: (1) hospitalized or living in nursing homes; (2) dying in the middle or withdrawing for reasons. The Jinzhou Medical University College of Nursing Ethics Committee gave its approval to the study (No. JZMULL2023062). Every older adult person who was recruited for the study was required to give written informed consent before taking part. G*Power 3.1 was used to calculate the sample size. The effect size was 0.15 (medium size), the significance level α was set at 0.05, and the statistical power (1-*β*) was 0.90. The minimum sample size was 183 cases. A total of 260 questionnaires were finally distributed.

### 2.2 Measurement

#### 2.2.1 General information questionnaire

The general information questionnaire was developed by searching the relevant literature and the clinical experience of the investigators. It covers data on disease-related as well as sociodemographic characteristics. Sociodemographic characteristics included gender, age, marital status, education level, medical insurance payment method, religion, status of residence, and household incomes *per capita* (yuan). Disease-related information comprised the duration of illness (year), number of comorbidities (kind), the type of disease, and self-care ability. Socio-demographic characteristics were collected by self-reporting, and part of the disease-related data (duration of illness, the type of disease) were filled in by the researcher through medical records.

#### 2.2.2 Home hospice care needs questionnaire for the dying older adult

The home hospice care needs questionnaire for the dying older adult (HHCNQ-DE) was an assessment tool developed by our research group to assess the home hospice care needs of the dying older adult in China. From April to September 2023, a total of 5 hospice pilot community health service centers were selected from 7 administrative districts in Jinzhou City, Liaoning Province by stratified sampling method. Secondly, a simple random sampling method was used to select a total of 199 terminally older adult people from the communities of pilot community service centers for psychological verification. The questionnaire had been psychologically validated and the instrument was found to have good reliability and validity. The Cronbach’s alpha coefficient of the total questionnaire was 0.832, the Cronbach’s alpha coefficients of the dimensions were 0.845–0.942, and the re-test reliability was 0.806. The content validity index of the questionnaire was 0.982, and the content validity index of the items was 0.83–1.00. The exploratory factors were extracted from the five common factors, and the cumulative variance contribution was 68.811%. The questionnaire included physiological needs (9 items), spiritual needs (7 items), psychological needs (6 items), social support needs (5 items) and information needs (4 items) 5 dimensions, a total of 31 items. Likert 5-point scale was used for each item, ranging from 1 to 5 points from “not needed” to “very needed.” The total questionnaire scores ranged from 31 to 155, with higher scores indicating higher levels of need. Among them, < 104 was defined as low need, 104–115 was defined as medium need, and > 115 was defined as high need.

### 2.3 Data collection

The household survey was carried out by the researcher as the investigator, and the community nurses in charge of home follow-up in each community health center assisted in the survey implementation. Before the survey, the purpose and significance of the study were explained to the respondents, and an informed consent form was signed. Then, we explained the requirements for filling out the questionnaire and asked the older adult to fill out the questionnaire on their own after they fully understood it. For those who were illiterate or physically weak, the researchers would read and explain the questionnaire item by item to the respondents, and finally record the survey results truthfully. The time limit for answering the questionnaire was 20 min. The survey was conducted at a time when the older adult had enough sleep and were not undergoing nursing care or treatment. Incomplete or single-option questionnaires were considered invalid.

### 2.4 Statistical analysis

Epidata 3.1 software was used for double entry of raw values and SPSS22.0 (IBM Corp, Armonk, NY) software was used for statistical analysis of data. The two-sided test level was α = 0.05, and the difference was statistically significant at *p* < 0.05.

General information about the study participants was described using frequency and component ratio, and the HHCNQ-DE total and dimension need scores were described using mean and standard deviation. To analyze differences, first, independent samples t-tests (variables grouped into two groups) and one-way ANOVA (variables grouped into three or more groups) were used to initially explore the relationship between the home hospice need dimension scores and general information about study participants. Second, using the HHCNQ-DE total score and the scores of the five dimensions as dependent variables, the statistically significant independent variables in the one-way analysis of variance were selected for multiple linear regression analyses (α _in_ = 0.05, α _out_ = 0.10) to find out the influencing factors of the total needs and the needs of the dimensions.

## 3 Results

### 3.1 Participant characteristics

A total of 260 questionnaires were distributed in this study, and 247 meeting the requirements were recovered, with a response rate of 95%. 13 invalid questionnaires were excluded, among which 9 refused to answer part of the questions in the questionnaire due to personal reasons, resulting in incomplete questionnaires. Four were excluded because the subjects filled in their errors.

More than half (53.80%) of the older adult dying of chronic diseases were women. The majority were 60–69 years old (40.50%), married or cohabiting (78.10%), with a primary school education or below (55.10%), using medical insurance for urban workers (53.40%), not practicing any religion (85.00%), and living with their spouses (63.20%). The most common type of disease was cancer (54.70%), of which lung cancer was more prevalent (40.00%). Among non-cancer chronic diseases, Cardiovascular diseases were more prevalent (40.18%). Table 1 displayed more general study population data.

**Table 1:** General information of survey participants (*N* = 247).

Characteristics		N	Percentage (%)
Gender	Male	133	53.80
	Female	114	46.20
Age (year)	60–69	100	40.50
	70–79	96	38.90
	≥80	51	20.60
Marital status	Unmarried	3	1.20
	Married/ Cohabitation	193	78.10
	Divorce	7	2.80
	Widowed	44	17.80
Education level	Primary school and below	136	55.10
	Middle school education	76	30.80
	High school education or technical secondary school	15	6.10
	Junior college and above	20	8.10
Medical insurance payment method	Insurance for urban workers	132	53.40
	New Rural Co-operative Medical System	94	38.10
	Commercial health insurance	6	2.40
	Self-paying	15	6.10
Religion	No	210	85.00
	Yes	37	15.00
Status of residence	Live with children	31	12.60
	Live with spouse	156	63.20
	Live alone	40	16.20
	Live with children and spouse	20	8.10
Duration of illness (year)	<1	81	32.80
	1–3	94	38.10
	>3	72	29.10
Household incomes *per capita* (yuan)	<1,500	93	37.70
	1,500–2,999	93	37.70
	3,000–4,999	46	18.60
	≥5,000	15	6.10
Number of Comorbidities (kind)	1	129	52.20
	2	81	32.80
	≥3	37	15.00
The type of disease	Non-cancer chronic diseases	112	45.30
	Cardiovascular diseases Chronic	45	18.23
	Respiratory diseases	32	12.94
	Nervous system diseases	15	6.07
	Cerebrovascular diseases	12	4.85
	Other	8	3.24
	Cancer	135	54.70
	Lung cancer	54	21.69
	Liver cancer	30	12.16
	Colorectal cancer	22	8.91
	Leukemia	12	4.86
	Esophageal cancer	10	4.05
	Other	7	2.84
Self-care ability	Completely capable of	67	27.10
	Most able to	96	38.90
	A part of	54	21.90
	Completely unable	30	12.10

### 3.2 Home hospice care needs score

The older adult with chronic diseases had a total score of 115.70 ± 12.79 for home hospice needs, with a mean score of 3.73 ± 0.41 for all items. The mean scores of the items of the dimensions of the needs in descending order were Information Needs (3.96 ± 0.61), Social Support Needs (3.96 ± 0.44), Spiritual Needs (3.92 ± 0.43), Physical Needs (3.60 ± 0.59), Psychological Needs (3.37 ± 0.65). More specific details are displayed in Table 2.

**Table 2:** Scores for items on dimensions of need for home hospice care for older adult with end-stage chronic disease (*N* = 247).

Dimension	Number of items	Range of score	Total score (X^−^ ± SD)	Items score (X^−^ ± SD)	Order
Physical Needs	9	9–45	32.40 ± 5.29	3.60 ± 0.59	4
Psychological Needs	6	6–30	20.19 ± 3.93	3.37 ± 0.65	5
Social Support Needs	5	5–25	19.82 ± 2.21	3.96 ± 0.44	2
Information Needs	4	4–20	15.83 ± 2.42	3.96 ± 0.61	1
Spiritual Needs	7	7–35	27.46 ± 3.03	3.92 ± 0.43	3
Total score	31	31–155	115.70 ± 12.79	3.73 ± 0.41	

The top and bottom five scoring items for each dimension are displayed in Table 3. The top 5 items were related to the dimensions of Information Needs and Social Support Needs, of which the top 2 need items were “Hope to get the care, concern and support of relatives and friends” (4.73 ± 0.48) and “I hope I can leave peacefully with my family by my side” (4.69 ± 0.51). The last 5 items in the ranking were primarily related to the Psychological Needs dimension, of which the last 2 need items were “Hope to resolve the contradictions and grievances between friends and relatives” (2.23 ± 1.06) and “I hope to help me regulate negative emotions such as anxiety and panic (such as fear of death)” (2.61 ± 1.16).

**Table 3:** Score for the first 5 and last 5 items of the need (N = 247).

	Item	Dimension	Score (X^−^ ± SD)
Top 5 items	Hope to get the care, concern and support of relatives and friends	Social support needs	4.73 ± 0.48
	I hope I can leave peacefully with my family by my side	Spiritual needs	4.69 ± 0.51
	It is hoped that medical staff provide long-term disease health guidance, empowerment education (such as pain assessment and mindfulness therapy), and assist family members to complete care	Social support needs	4.48 ± 0.77
	It is hoped that the community will carry out pair assistance (such as social workers and volunteers to provide care services) and mutual assistance activities (such as peer support for the older adult)	Social support needs	4.33 ± 0.79
	Hope to respect my end-of-life medical decisions (such as refuse invasive treatment)	Spiritual needs	4.30 ± 0.78
Last 5 items	Hope to resolve the contradictions and grievances between friends and relatives	Social support needs	2.23 ± 1.06
	I hope to help me regulate negative emotions such as anxiety and panic (such as fear of death)	Psychological needs	2.61 ± 1.16
	Hope to help me regulate the negative emotions of depression and pessimism (e.g., due to weakness, illness and pain)	Psychological needs	2.72 ± 1.21
	Hope to respect my privacy and dignity	Spiritual needs	3.29 ± 0.99
	Hope you can help me with daily care (such as skin care, oral care)	Physical needs	3.32 ± 0.94

### 3.3 Univariate analyses of the home hospice care needs

In Table 4, there were significant differences in the home care needs of older adults with end-stage chronic disease based on several sociodemographic and disease-related factors. Regarding the overall needs score, the dying older adult with older age, lower education, commercial health insurance, religious beliefs, living with children and spouse, long illness duration, monthly household income of 3,000–4,999 yuan, many chronic illnesses, non-cancer chronic disease, and partial able to care had higher HHCNQ-DE scores.

**Table 4:** Univariate analysis of home hospice care needs (independent samples t-test or one-way analysis of variance, x̄ ±s).

Variable		Physical needs	Psychological needs	Social support needs	Information needs	Spiritual needs	Total score
Gender	Female	32.51 ± 5.13	20.20 ± 3.85	19.96 ± 2.19	16.00 ± 2.28	27.76 ± 2.81	116.43 ± 11.85
	Male	32.26 ± 5.50	20.19 ± 4.03	19.96 ± 2.24	15.54 ± 2.57	27.11 ± 3.25	114.85 ± 13.81
Age (year)	60–69	***30.91 ± 5.02***	20.01 ± 4.02	***19.28 ± 2.27***	15.91 ± 2.08	27.02 ± 2.73	***113.13 ± 12.53***
	70–79	***32.35 ± 5.18***	20.50 ± 4.04	***20.19 ± 2.16***	15.75 ± 2.61	27.85 ± 3.14	***116.65 ± 12.88***
	≥80	***35.39 ± 4.84***	19.98 ± 3.54	***19.98 ± 3.54***	15.84 ± 2.69	27.57 ± 3.32	***118.96 ± 12.37***
Marital status	Unmarried	***37.33 ± 1.53***	21.33 ± 1.16	22.00 ± 0.00	16.67 ± 3.22	27.00 ± 0.00	124.33 ± 2.31
	Married/Cohabitation	***31.92 ± 5.27***	20.29 ± 4.21	19.68 ± 2.22	15.89 ± 2.39	27.56 ± 3.14	115.35 ± 13.32
	Divorced	***30.43 ± 5.47***	17.43 ± 2.44	19.71 ± 1.70	15.57 ± 2.76	26.14 ± 2.73	109.29 ± 11.74
	Widowed	***34.45 ± 4.96***	20.14 ± 2.57	20.27 ± 2.22	15.59 ± 2.51	27.23 ± 2.68	117.68 ± 10.87
Education level	Primary school and below	***33.7 ± 5.15***	***20.63 ± 3.60***	***20.37 ± 2.05***	***16.07 ± 2.33***	***27.94 ± 3.03***	***118.7 ± 11.75***
	Middle school education	***29.82 ± 4.87***	***19.13 ± 4.40***	***18.78 ± 2.38***	***15.09 ± 2.71***	***26.3 ± 3.00***	***109.12 ± 13.52***
	High school education or technical secondary school	***31.87 ± 5.25***	***19.8 ± 3.53***	***19.87 ± 1.46***	***16.4 ± 1.24***	***27.33 ± 2.06***	***115.27 ± 8.84***
	Junior college and above	***33.75 ± 4.45***	***21.6 ± 3.69***	***20 ± 1.72***	***16.65 ± 1.87***	***28.65 ± 2.54***	***120.65 ± 9.72***
Medical insurance payment method	Insurance for urban workers	***32.86 ± 5.06***	19.97 ± 3.89	19.96 ± 2.30	***16.17 ± 2.32***	***27.76 ± 3.09***	***116.72 ± 12.37***
	New Rural Co-operative Medical System	***31.00 ± 5.49***	20.24 ± 4.15	19.47 ± 2.08	***15.36 ± 2.51***	***26.71 ± 2.95***	***112.79 ± 13.39***
	Commercial health insurance	***35.33 ± 3.33***	24.00 ± 3.69	21.67 ± 1.63	***14.17 ± 1.84***	***29.33 ± 2.58***	***124.5 ± 9.33***
	Self-paying	**35.87 ± 4.19**	20.33 ± 1.72	20.00 ± 2.10	***16.53 ± 2.36***	***28.73 ± 1.98***	***121.47 ± 9.13***
Religion	No	***31.90 ± 5.21***	20.18 ± 3.87	19.71 ± 2.20	***15.70 ± 2.41***	27.28 ± 2.82	***114.77 ± 12.28***
	Yes	***35.22 ± 4.92***	20.27 ± 4.30	20.43 ± 2.24	***16.57 ± 2.36***	28.49 ± 3.93	***120.97 ± 14.43***
Status of residence	Live with children	***32.84 ± 5.69***	***19.9 ± 3.02***	***19.71 ± 2.15***	***19.71 ± 2.53***	***26.71 ± 3.04***	***113.74 ± 13.02***
	Live with spouse	***31.08 ± 5.06***	***19.74 ± 3.89***	***19.40 ± 2.27***	***19.40 ± 2.46***	***27.01 ± 2.82***	***112.97 ± 12.12***
	Live alone	***34.93 ± 3.63***	***21.05 ± 3.01***	***20.93 ± 1.73***	***20.93 ± 1.88***	***28.18 ± 2.22***	***121.5 ± 7.00***
	Live with children and spouse	***36.9 ± 5.14***	***22.45 ± 5.89***	***21.00 ± 1.52***	***21.00 ± 1.87***	***30.70 ± 3.83***	***128.4 ± 15.60***
Duration of illness (year)	<1	***29.94 ± 5.32***	***19.30 ± 4.49***	***19.09 ± 2.36***	***15.49 ± 2.44***	26.85 ± 3.03	***110.67 ± 14.40***
	1–3	***32.4 ± 4.73***	***20.96 ± 3.84***	***20.04 ± 2.14***	***15.70 ± 2.76***	27.69 ± 3.19	***116.80 ± 11.95***
	>3	***35.15 ± 4.62***	***20.21 ± 3.12***	***20.35 ± 1.94***	***16.39 ± 1.77***	27.83 ± 2.75	***119.93 ± 9.87***
Household incomes *per capita* (yuan)	<1,500	31.69 ± 5.64	***20.52 ± 3.93***	***19.44 ± 2.09***	15.52 ± 2.69	***27.11 ± 2.99***	***114.27 ± 13.66***
	1,500–2,999	32.30 ± 4.88	***18.97 ± 3.28***	***19.71 ± 2.28***	15.74 ± 2.31	***26.89 ± 2.73***	***113.61 ± 10.75***
	3,000–4,999	33.76 ± 5.61	***22.00 ± 4.55***	***20.54 ± 2.12***	16.35 ± 2.18	***29.15 ± 3.32***	***121.80 ± 14.26***
	≥5,000	33.20 ± 3.99	***20.27 ± 3.31***	***20.60 ± 2.29***	16.8 ± 1.57	***27.93 ± 2.40***	***118.80 ± 7.70***
Number of Comorbidities (kind)	1	***30.71 ± 5.26***	19.94 ± 4.18	19.57 ± 2.17	15.78 ± 2.47	27.35 ± 2.96	***113.68 ± 13.32***
	2	***33.49 ± 4.88***	20.52 ± 3.64	19.79 ± 2.15	15.94 ± 2.46	27.74 ± 3.25	***117.48 ± 12.64***
	≥3	***35.86 ± 3.90***	20.38 ± 3.64	19.91 ± 2.28	15.81 ± 2.18	27.22 ± 2.80	***118.84 ± 10.01***
The type of disease	Non-cancer chronic diseases	***35.30 ± 4.00***	20.37 ± 3.23	***20.81 ± 1.81***	***16.25 ± 2.30***	***28.23 ± 2.95***	***120.96 ± 9.97***
	Cancer	***29.99 ± 3.00***	20.05 ± 4.43	***18.99 ± 2.18***	***15.49 ± 2.47***	***26.81 ± 2.96***	***111.33 ± 13.25***
Self-care ability	Completely capable of	***29.39 ± 5.40***	19.04 ± 4.50	***19.30 ± 1.88***	15.94 ± 2.19	27.28 ± 3.38	***110.96 ± 13.94***
	Most able to	***32.26 ± 5.09***	20.6 ± 3.91	***19.85 ± 2.37***	15.99 ± 2.46	27.86 ± 2.83	***116.57 ± 12.61***
	A part of	***34.52 ± 4.06***	20.8 ± 3.10	***20.65 ± 1.99***	15.98 ± 2.37	27.41 ± 2.86	***119.35 ± 10.00***
	Completely unable	***35.73 ± 3.95***	20.37 ± 3.56	***19.37 ± 2.39***	14.83 ± 2.74	26.63 ± 3.09	***116.93 ± 12.68***

The bold and italics indicated the *p* < 0.05.

### 3.4 Multiple linear regression analysis of factors influencing the need for home hospice care

The independent variables medical insurance payment method (based on Insurance for urban workers) and status of residence (based on living with children) were set as dummy variables. Other independent variables were assigned values, like age, which was coded as 1 for 69–69 years, 2 for 70–79 years, and 3 for ≥80 years. The independent variable assignments and dummy variable settings are shown in Table 5.

**Table 5:** Dummy variable setting and independent variable assignment situation.

Variable	Assignment mode			
Age (year)	60–69 = 1	70–79 = 2	≥80 = 3	
Education level	Primary school and below = 1	Middle school education = 2	High school education or technical secondary school = 3	Junior college and above = 4
Medical insurance payment method	Insurance for urban workers = 0	New Rural Co-operative Medical System = 1	Commercial health insurance = 0	Self-paying = 1
	Insurance for urban workers = 0	New Rural Co-operative Medical System = 0	Commercial health insurance = 1	Self-paying = 0
	Insurance for urban workers = 0	New Rural Co-operative Medical System = 0	Commercial health insurance = 0	Self-paying = 1
Religion	No = 1	Yes = 2		
Status of residence	Live with children = 0	Live with spouse = 1	Live alone = 0	Live with children and spouse = 1
	Live with children = 0	Live with spouse = 0	Live alone = 1	Live with children and spouse = 0
	Live with children = 0	Live with spouse = 0	Live alone = 0	Live with children and spouse = 1
Duration of illness (year)	<1 = 1	1–3 = 2	>3 = 3	
Household incomes *per capita* (yuan)	<1,500 = 1	1,500–2,999 = 2	3,000–4,999 = 3	≥5,000 = 4
Number of Comorbidities (kind)	1 = 1	2 = 2	≥3 = 3	
The type of disease	Non-cancer chronic diseases = 1	Cancer = 2		
Self-care ability	Completely capable of = 1	Most able to = 2	A part of = 3	Completely unable = 4

Six multivariate linear regression analyses were performed to identify significant correlates of overall home hospice needs and five dimensions of needs (see Table 6). The diagnostic test independent variables had all variance inflation factor (VIF) values less than 10, indicating no multicollinearity issue existed before the analysis was conducted. Furthermore, residual analysis provided support for the equation models’ linearity, normality, and homogeneity of variance. The terminal older adult lived with children and spouse (*β* = 16.385, *p* = 0.000), had a disease duration ≥3 years (*β* = 2.721, *p* = 0.007), had a non-cancer chronic disease (*β* = −5.874, *p* = 0.002), and were completely unable to care for themselves (*β* = 1.919, *p* = 0.031) may have a higher total need for home hospice care, with 30% of the variance explained. The terminal older adult who used commercial health insurance to pay for health care (*β* = 3.808, *p* = 0.031), lived with children and spouse (*β* = 5.566, *p* = 0.000), had a disease duration ≥3 years (*β* = 1.154, *p* = 0.001), had a comorbid combination of ≥3 chronic diseases (*β* = 1.302, *p* = 0.001), with non-cancer chronic diseases (*β* = −5.874, *p* = 0.000), and completely unable to care for themselves (*β* = 1.463, *p* = 0.031) were more likely to have Physical Needs, explaining 49.6% of the variance. The terminal older adult used commercial medical insurance to pay medical expenses (*β* = 4.384, *p* = 0.031), lived with children and spouse (*β* = 3.925, *p* = 0.000) and were completely unable to take care of themselves (*β* = 0.814, *p* = 0.008) were significantly correlated with Psychological Needs, which explained 11.4% of the variance. Duration of illness ≥3 years (*β* = 0.421, *p* = 0.019), comorbidity of ≥3 chronic disease (*β* = −0.561, *p* = 0.003), and suffering from non-cancer chronic disease (*β* = −1.537, *p* = 0.000) were higher in the terminally ill older adult Social Support Needs, explaining 25.5% of the variance. The use of urban workers’ insurance to pay for medical expenses (*β* = −2.925, *p* = 0.005) and living with children and spouse (*β* = 2.145, *p* = 0.003) among the terminally ill older adult were significantly associated with Information Needs, explaining 17.2% of the variance. Terminally ill older adult were more likely to express Spiritual Needs by paying for healthcare out-of-pocket (*β* = 1.689, *p* = 0.043), living with children and spouse (*β* = −3.551, *p* = 0.000), and having a non-cancer chronic disease (*β* = −1.001, *p* = 0.037), explaining 19.5% of the variance.

**Table 6:** Multiple linear regression analysis of total home hospice need and factors influencing need by dimension.

Dependent variable	Independent variable	Unstandardized coefficients		Standardized coefficients	*R*^2^	Adjusted *R*^2^	*t*	*p*-value
		*β*	SE	*β*				
Total score	(Constant)	107.167	6.894		0.3	0.258	15.545	0
	Status of residence							
	Live alone	5.594	2.817	0.161			1.985	0.048
	Live with children and spouse	16.385	3.464	0.35			4.73	0
	Duration of illness (year)	2.721	0.994	0.168			2.739	0.007
	The type of disease	−5.874	1.878	−0.229			−3.128	0.002
	Self-care ability	1.919	0.883	0.146			2.174	0.031
Physical needs	(Constant)	27.262	2.423		0.496	0.465	11.253	0.000
	Medical insurance payment method							
	Commercial health insurance	3.808	1.758	0.111			2.166	0.031
	Self-paying	2.514	1.144	0.114			2.197	0.029
	Status of residence							
	Live with children and spouse	5.566	1.217	0.287			4.573	0.000
	Duration of illness (year)	1.154	0.349	0.172			3.306	0.001
	Number of Comorbidities (kind)	1.302	0.373	0.18			3.488	0.001
	The type of disease	−3.028	0.66	−0.285			−4.589	0.000
	Self-care ability	1.463	0.31	0.268			4.716	0.000
Psychological needs	(Constant)	15.897	2.381		0.114	0.061	6.715	0.000
	Medical insurance payment method							
	Commercial health insurance	4.348	1.727	0.171			2.518	0.012
	Status of residence							
	Live with children and spouse	3.925	1.196	0.273			3.282	0.001
	Self-care ability	0.814	0.305	0.201			2.671	0.008
Social support needs	(Constant)	21.397	1.231		0.255	0.21	17.384	0
	Duration of illness (year)	0.42	0.177	0.149			2.366	0.019
	Number of Comorbidities (kind)	0.561	0.19	0.186			2.959	0.003
	The type of disease	−1.537	0.335	−0.346			−4.584	0.000
Information needs	(Constant)	15.281	1.419		0.172	0.122	10.716	0.000
	Medical insurance payment method							
	Commercial health insurance	−2.925	1.03	−0.186			−2.84	0.005
	Status of residence							
	Live with spouse	1.057	0.475	0.211			2.224	0.027
	Live alone	1.777	0.58	0.271			3.063	0.002
	Live with children and spouse	2.145	0.713	0.242			3.008	0.003
Spiritual needs	(Constant)	27.315	1.735		0.195	0.146	15.578	0
	Medical insurance payment method							
	Self-paying	1.689	0.828	0.133			2.04	0.043
	Status of residence							
	Live with children and spouse	3.551	0.881	0.32			4.03	0.000
	The type of disease	−1.001	0.487	−0.165			−2.096	0.037

## 4 Discussion

The results of this study showed that the total score for home hospice needs was 115.70 ± 12.79, indicating that there is a high level of hospice needs for the older adult at the end of life with chronic diseases in the home setting. The highest scoring items were Information Needs, Social Support Needs, and Spiritual Needs in that order, indicating that these three dimensions are of particular concern to the older adult dying at home in China, and that there are still many unmet needs in these three dimensions with the existing medical resources. The three items with higher scores in Social Support Needs implied that the dying older adult longed for assistance from family members, medical staff, social workers, and senior peers, which was similar to the results of Hu et al. (24). The item that received the lowest score in terms of Social Support Needs was “Hope to resolve the contradictions and grievances between friends and relatives,” which suggests that the dying older adult in this study had harmonious relationships with their friends and relatives. Dunham et al. believed that harmonious interpersonal relationships could help older adult cancer patients enhance their sense of self-worth and reduce psychological anxiety (25). Two Spiritual Needs items with higher scores expressed the dying older adult’s need for increased autonomy. Since the older adult are respected in their right to die at home and have essentially had their wish to pass away with dignity fulfilled, the low score for “Hope to respect my privacy and dignity” can be explained.

The dimensions of physical and psychological needs had the lowest mean scores among the items. Ghesquiere demonstrated that older people who had multiple chronic illnesses experienced more depressive feelings along with a greater number of physical symptoms (e.g., pain, and fatigue) (26). Therefore, it is not implied that these two dimensions were met by the study’s low results. The low scores may be because China is deeply influenced by Confucian culture, which is family-centered, and not being a burden to the family is the greatest expectation of the dying older adult (27), which is reflected in the item “Hope to help me with daily care (such as skin care, oral care).” Family members are responsible for personal care, symptom management, and emotional support for the dying older adult, and the responsibility of caring for them makes the caregiver’s burden of care heavy. In order to minimize the burden and indebtedness to the family, the dying older adult conceal their actual feelings and tolerate their physical discomfort.

Next, we determined a number of variables pertaining to the need for home hospice care for the older adult dying of chronic illness. The study’s findings demonstrated that the status of residence was an influential factor of needs, with those who live with their spouse and children reporting higher levels of physical, psychological, informational, and spiritual needs. Contrary to the results of several scholars (28, 29). One potential explanation could be the low monthly household income of the study’s population, of which 75.4% earn less than ≤3,000 yuan per month. This amount of money is far less than the monthly *per capita* income of Liaoning Province’s urban residents in 2022 (3,667 yuan) (30). Higher costs are associated with more people living together, and older people’s access to high-quality healthcare is restricted by the state of the economy. At present, home hospice care is not covered in its whole by medical insurance in China (31). Due to financial constraints, older patients who are terminally ill only accept a portion of the home hospice care service program, so the fact cannot meet their basic physiological needs. According to Maslow’s Hierarchy of Needs Theory, unmet physiological needs make it difficult to meet other needs at a higher level (32). Furthermore, this study discovered that due to differences in reimbursement rates, different types of insurance are linked to various needs. In order to relieve financial strain, it is advised that healthcare professionals help family members apply for grants and benefits, as well as applications for donations from individuals and social organizations for the older adult with terminal illnesses. China needs to reform its health insurance system in the future, intending to break down the barriers of the home hospice medical system and provide institutional safeguards for the development of the home hospice care business.

In this study, end-of-life older adult with longer illness duration and more comorbidities had higher physical and social support needs, which was consistent with the findings of Wyld et al. (33). The reason for this analysis is the progressive aggravation of symptoms brought about by the disease as the duration of the disease increases. Studies have shown that cancer patients with longer disease duration experience more symptoms than patients with shorter disease duration (28), which suggests that the duration of the disease is closely related to the patient’s quality of life. Relevant studies have found that heart failure is often complicated by diabetes mellitus, arrhythmia, hypertension and other diseases (34). The combination of chronic diseases in older adult patients can cause multi-system damage, leading to a decline in physiologic function, increasing the burden of medication (increased adverse drug reactions, decreased medication adherence), and severely reducing the quality of life. Wang et al. have found that a higher level of social support can positively affect patients (35). Support from healthcare professionals in relation to physical symptoms and moral support from family and friends ultimately improved their quality of life. Consequently, healthcare professionals should provide early home hospice care for the older adult chronic population can effectively reduce disease distress. Simultaneously, we should highlight the function and influence of social support on older persons with chronic illnesses and their families, as well as develop practical strategies to offer social interventions to patients.

According to the study’s findings, older adult with non-cancer chronic diseases had higher home hospice care needs than those with cancer, particularly in terms of their physical, social, and spiritual needs. This agrees with Llop-Medina et al.’s (36) findings. Theoretical trajectories of dying of patients with advanced cancer, proposed by Glaser and Strauss, can predict changes in the condition of cancer patients in advance (37). Healthcare professionals can make appropriate end-of-life care decisions based on changes in symptoms. In contrast, non-cancer-diagnosed patients are characterized by long-term, progressive and rapid deterioration (38), and the difficulty in grasping the course of the disease makes it more difficult to carry out nursing care. Burt believes that it is difficult to meet the physiological needs of the patient without effective symptom management or adequate nursing care (39). Repeated changes in disease not only increase physical pain but also mental suffering and a diminished sense of confidence and meaning in life. Roughly half of patients with severe heart failure will pass away suddenly from sudden cardiac death (40); this suggests that a professional hospice team is necessary due to the disease’s rapid deterioration. Therefore, community healthcare professionals should encourage seniors and their families to develop advanced care planning as early as possible to cope with the unpredictability of chronic disease at the end of life. It is also possible to establish a hospice care multidisciplinary chronic disease specialist team, according to different types of chronic diseases, through division of labor and cooperation, to improve the sense of meaning in life based on relieving physical pain, and provide timely professional help when the disease deteriorates rapidly.

This study’s findings, which are in line with those of Kun Zhang and Chaoying Wang’s (28, 29) study, show that the poorer the self-care ability of the terminal older adult, the higher the need for home hospice care, especially physical needs and psychological needs. With the increase of age, the self-care ability of the older adult gradually decreases. According to the current survey, 72.9% of older persons have defective self-care ability, which means that they greatly depend on the assistance of others. Influenced by the traditional Chinese concept of “raising children to prevent old age,” most of the dying older adult tend to choose family members to provide care for them (41). Due to the lack of caregiving knowledge, it is difficult for family members to manage their symptoms effectively while providing basic care such as daily living. For instance, Tarter’s research revealed that informal caregivers were inefficient in managing dementia patients’ pain (42). In addition, dying older adult have restricted activities due to their illness (dyspnea, heart failure), which often keeps them indoors and isolates them from the outside world. This creates psychological needs like loneliness. As a result, community medical staff use both online and offline techniques to instruct caregivers on caregiving skills. If the condition permits, assist family members in transferring the patient to the outdoor area to talk with others and participate in social activities together, to reduce the sense of social alienation; When the illness does not allow outdoor activities, assist family members in arranging home activities, such as having dinner together or accompanying the patient to catch up with old times.

## 5 Limitations

The following are the limitations of this study. First, the classification of chronic illness categories and comorbidities was overly simple and rough due to the small sample size. To determine the differences in need, the classification of chronic diseases can be further differentiated in the future using a sizable sample size. Second, this study was conducted in Jinzhou City, Liaoning Province, which is useful for studies under the same economic and cultural conditions, but whether the conclusions are representative of the chronic terminal older adult population in other regions needs to be further explored. Future studies can collect data in different regions to make the findings more generalizable. Finally, this study was a cross-sectional survey that investigated the need for and factors influencing home hospice care for this population at a certain point in time. In the future, a longitudinal study could be conducted to investigate trends in home hospice needs and influencing factors as their condition changes. Based on this study, we need to continue to explore cultural differences in hospice needs at home or evaluate the effectiveness of different hospice interventions in the future.

## 6 Conclusion

In summary, the need for home hospice care in China is high for the older adult with end-stage chronic disease, who need care in physical, psychological, spiritual, and social support. Among them, status of residence, duration of illness (year), the type of disease, and self-care ability were the main factors influencing the need for home hospice care. In the process of providing hospice care services for the dying older adult and their families, healthcare professionals should assess their needs and fully consider the influencing factors of needs, so as to promote the improvement of the level of hospice care services. Moreover, the provision of personalized and comprehensive care and nursing care centered on the dying older adult can improve their quality of life within a limited period of time. It is also necessary for government departments to standardize and formulate a comprehensive hospice policy so that older adult patients can finish their final journey in a home environment, quietly and comfortably.

## Data availability statement

The original contributions presented in the study are included in the article/supplementary material, further inquiries can be directed to the corresponding author.

## Ethics statement

The studies involving humans were approved by Jinzhou Medical University College of Nursing Ethics Committee. The studies were conducted in accordance with the local legislation and institutional requirements. The participants provided their written informed consent to participate in this study.

## Author contributions

LW: Writing – original draft. YL: Writing – original draft. RZ: Writing – review & editing. JL: Writing – review & editing. XG: Writing – original draft. HL: Writing – review & editing. YC: Writing – review & editing.
